# Challenges and Solutions in Postoperative Complications: A Narrative Review in General Surgery

**DOI:** 10.7759/cureus.50942

**Published:** 2023-12-22

**Authors:** Herra Javed, Olusegun A Olanrewaju, Frank Ansah Owusu, Ayesha Saleem, Peddi Pavani, Humza Tariq, Brigitte Soledad Vasquez Ortiz, Raja Ram, Giustino Varrassi

**Affiliations:** 1 General Surgery, Shifa College of Medicine, Islamabad, PAK; 2 Pure and Applied Biology, Ladoke Akintola University of Technology, Ogbomoso, NGA; 3 General Medicine, Stavropol State Medical University, Stavropol, RUS; 4 Medicine, Stavropol State Medical University, Stavropol, RUS; 5 General Surgery, Hayatabad Medical Complex (HMC), Peshawar, PAK; 6 General Surgery, Kurnool Medical College, Kurnool, IND; 7 Surgery, Lahore General Hospital, Lahore, PAK; 8 General Surgery, Universidad del Azuay, Cuenca, ECU; 9 Medicine, MedStar Washington Hospital Center, Washington, USA; 10 Pain Medicine, Paolo Procacci Foundation, Rome, ITA

**Keywords:** postoperative complications, surgical interventions, general surgery, complications, postoperative

## Abstract

In general surgery, the goal of achieving favorable results following surgical procedures is consistently impeded by the intricate range of postoperative problems. This abstract summarizes a comprehensive narrative study that examines the numerous difficulties associated with postoperative complications and investigates potential remedies. With the progress of surgical practices, the intricacies of complications also increase, requiring a flexible comprehension of the diverse scenarios. This review examines the many factors contributing to postoperative complications, including patient-specific variables and advancing surgical procedures. It also explores the broader consequences of these problems on individual patients and healthcare systems. The economic results, such as extended hospitalizations and increased allocation of resources, highlight the need for specific solutions. This abstract also emphasizes the review's examination of novel methodologies, technology incorporations, and cooperative tactics as potential transformative factors. This abstract provides an overview of the ongoing efforts to change how postoperative complications are understood in general surgery. It highlights the importance of taking preventive measures and adopting a comprehensive approach to patient care.

## Introduction and background

Surgical treatments are crucial in treating various medical diseases, including standard and complicated instances. Although surgical procedures have made significant progress, the possibility of postoperative complications remains a considerable concern, negatively impacting the overall upward trend of surgical outcomes [1]. Gaining a comprehensive understanding of the complexities of these problems is crucial, not only for providing optimal care to individual patients but also for guiding the development of broader healthcare initiatives. This review examines the difficulties presented by postoperative complications in general surgery. It investigates possible solutions that could lead to enhanced patient care [2]. The complex interaction of several elements, such as patient-specific variables, surgical procedures, and complexities within the healthcare system, defines the landscape of postoperative complications. To fully comprehend the reasons behind investigating postoperative complications in general surgery, it is essential to explore these occurrences' complex and multifaceted nature [3]. The growing intricacy of surgical techniques and the expanding demographic variety of patients receiving surgery highlight the crucial necessity for a detailed analysis of postoperative problems.

Furthermore, the significant financial cost linked to postoperative complications should be considered. Healthcare systems worldwide struggle with the cost consequences of extended hospital stays, readmissions, and the extra resources needed to handle post-surgical problems [4]. Hence, the purpose of this narrative review goes beyond the clinical domain to aid in formulating methods that can reduce the human and financial burdens linked to postoperative problems in general surgery [5]. The importance of postoperative complications in general surgery is complex, as they impact patient morbidity, mortality, and the overall quality of healthcare provision. In addition to directly harming individual patients, these consequences have ripple effects on the healthcare system, influencing how resources are allocated, healthcare costs, and the overall surgical environment [5].

Postoperative complications significantly jeopardize patient outcomes, potentially leading to extended hospitalization, heightened reliance on healthcare resources, and an elevated likelihood of morbidity and mortality [6]. Surgical procedures intended to relieve pain and enhance well-being can paradoxically become causes of distress when complications occur. Hence, comprehending the importance of postoperative complications is essential for improving preoperative risk assessments, optimizing surgical procedures, and customizing postoperative care plans [7]. Postoperative problems have a significant impact on the economic aspects of healthcare when seen from a systemic standpoint. The cost consequences of dealing with complications, including prolonged hospital stays, extra medical procedures, and the possibility of being readmitted, highlight the urgent need to create preventive measures and focused treatments [8]. Moreover, the growing focus on value-based care underscores the necessity of reducing postoperative complications as healthcare systems globally strive to improve efficiency and quality. The investigation of difficulties and remedies for postoperative complications in the field of general surgery is more than just a scholarly endeavor. This initiative is based on a solid dedication to improving patient care, maximizing healthcare resources, and enhancing the core aspects of surgical practice [9]. This narrative review aims to enhance the ongoing discussion surrounding general surgery by thoroughly understanding postoperative complications. It emphasizes reducing these complications to benefit individual patients and the healthcare system.

## Review

Methodology

The methodology for conducting a narrative review on "Challenges and Solutions in Postoperative Complications" entails a systematic approach that adheres to the Scale for the Assessment of Narrative Review Article (SANRA) principles. This procedure involves the creation of a thorough search strategy, establishing precise criteria for including and excluding information, and a careful process of extracting data.

Search Strategy

A systematic search method will be utilized to examine the current body of literature thoroughly. The search will encompass multiple academic databases, such as PubMed, Scopus, and Google Scholar. The selection and combination of keywords and Medical Subject Headings (MeSH) terms pertaining to postoperative problems will be done meticulously to optimize the retrieval of pertinent articles. The search will be refined, and a balance between sensitivity and specificity will be ensured by using Boolean operators (AND, OR).

Inclusion and Exclusion Criteria

The inclusion criteria will be established to specifically target research directly addressing the difficulties and remedies associated with postoperative complications. Only scholarly works that have undergone peer review, systematic reviews, meta-analyses, and observational studies published within the past 10 years will be considered. Non-English studies or studies that are not relevant to the specific emphasis of the review will be omitted. To ensure the review's integrity, the inclusion and exclusion criteria will be uniformly applied to all identified papers.

Data Extraction

A consistent data extraction form will be created to retrieve relevant information from chosen articles. The data extraction procedure would encompass specific information, like research design, sample size, patient demographics, types of postoperative issues addressed, and potential treatments or interventions. In addition, we will collect data about the technique employed in each study, as well as the essential findings and limitations. The systematic methodology for extracting data will simplify the process of combining information and allow for a thorough examination of the difficulties and resolutions related to postoperative problems.

Quality Assessment

An evaluation will be performed to examine the quality of the studies presented to determine the evidence's reliability and validity. The methodological rigor of each study will be evaluated using a recognized instrument, such as the Joanna Briggs Institute critical appraisal tools for different study designs. This stage is crucial for guaranteeing the legitimacy and relevance of the findings included in the narrative review.

Synthesis of Findings

The narrative synthesis will systematically arrange the acquired data and highlight significant themes, patterns, and trends about the difficulties and resolutions associated with postoperative problems. The synthesis will be executed systematically and rationally, providing a lucid narrative progression that allows readers to comprehend the present state of understanding regarding the subject. This narrative review offers a thorough and detailed analysis of the difficulties and remedies associated with postoperative complications by following the SANRA guidelines and utilizing a meticulous methodology.

Types of postoperative complications in general surgery

Postoperative complications in general surgery refer to a wide range of difficulties that might occur after surgical procedures. These problems can substantially impact patient outcomes, lengthen the recovery process, and present extra dangers to general health [1]. This discussion will explore many categories of postoperative complications in general surgery, encompassing wound healing, infections, bleeding and hemorrhage, organ dysfunction, and other pertinent consequences.

Wound Healing Issues

The process of wound healing is intricate and can be hindered by multiple circumstances, resulting in problems. Surgical site infections (SSIs) are a significant issue characterized by erythema, edema, and purulent exudate at the incision site [2]. Inadequate wound healing can lead to dehiscence, the partial or total separation of the wound margins. This can heighten the likelihood of infections and hinder the healing process. Impaired wound healing is caused by factors such as limited blood flow, underlying medical problems, and poor postoperative care [3].

Infections

Postoperative infections substantially cause illness and death in general surgery. Surgical operations break the skin barrier, providing a pathway for diseases to enter. Furthermore, deep-seated infections can arise at the surgical site or in distant organs alongside SSIs [4]. Systemic manifestations of infection, such as pyrexia and increased leukocyte count, may suggest a more infectious severe consequence. Timely recognition, suitable antibiotic treatment, and, in certain instances, surgical intervention are essential for adequately controlling postoperative infections.

Bleeding and Hemorrhage

Bleeding and hemorrhage are severe complications that may arise during or after surgery, presenting a direct and life-threatening risk to the patient. Surgical procedures entail the incision of blood vessels, and although thorough hemostasis is a customary technique, bleeding may still ensue [5]. Postoperative bleeding occurring immediately after surgery may result in a hematoma, a localized accumulation of blood that can affect nearby tissues. Delayed bleeding, which arises from hours to a few days following a surgical procedure, may suggest the presence of vascular damage, coagulation disorders, or infection. Swift intervention, typically involving surgical examination and steps to stop bleeding, is crucial for effectively managing bleeding problems [6].

Organ Dysfunction

General surgery involves procedures on multiple organs, and difficulties may occur when these organs do not operate effectively after surgery. Reduced lung expansion following surgery can lead to respiratory problems, such as atelectasis and pneumonia. Patients with preexisting cardiovascular problems may experience cardiac consequences, such as arrhythmias and myocardial infarction [7]. Renal dysfunction, marked by the sudden impairment of kidney function, can arise from causes such as reduced blood flow to the kidneys and the use of drugs that are toxic to the kidneys. Continuous surveillance and proper handling are crucial to avoid and control organ dysfunction. Additional difficulties may occur throughout the postoperative period, in addition to the ones discussed earlier [8]. Immediate intervention is necessary for adverse reactions to anesthesia, such as respiratory depression and allergic responses. Thromboembolic events, such as deep vein thrombosis and pulmonary embolism, are hazardous for patients who are undergoing major surgery. Neurological problems, including delirium and nerve damage, can arise, underscoring the significance of doing a neurologic evaluation in the postoperative environment [9]. Metabolic problems, such as disturbances in electrolyte levels and high blood sugar levels, require close monitoring to maximize the patient's recovery.

Postoperative complications in the field of general surgery are diverse and have a substantial impact on the overall well-being of patients. Many complications, such as wound healing problems, infections, bleeding and hemorrhage, organ dysfunction, and other concerns, highlight the intricacy of surgical procedures [9]. Ensuring positive surgical outcomes requires being vigilant in perioperative care, adhering to evidence-based practices, and promptly recognizing and managing problems. Ongoing research and progress in surgical techniques and perioperative care protocols help reduce the occurrence and severity of postoperative issues in the ever-evolving area of general surgery [10].

Prevalence and incidence rates

Postoperative complications are a significant concern in the field of surgery, as they have a considerable impact on patient outcomes and the allocation of healthcare resources. Comprehending these consequences' frequency and occurrence rates is vital for healthcare practitioners, researchers, and policymakers to establish efficient preventive measures and enhance patient care [11]. This discussion presents an epidemiological analysis of postoperative complications, examining their prevalence, incidence rates, and related risk factors.

Epidemiological Overview

The field of postoperative complications epidemiology focuses on examining the prevalence and causes of these problems among a specific population [11]. Most postoperative problems are the ratio of individuals who experience difficulties to the total number of individuals who have undergone surgical operations. Incidence rates, however, indicate the frequency of new occurrences within a defined time frame [12]. These epidemiological metrics offer valuable information on the prevalence of postoperative problems and help inform and address prevention efforts.

Prevalence and Incidence Rates

The frequency and occurrence of postoperative problems differ among surgical techniques, patient groups, and healthcare environments. Evidence repeatedly demonstrates that a significant fraction of individuals undergoing surgery encounter postoperative complications, which can range from minor problems to adverse severe occurrences [13]. Factors such as the intricacy of the surgery, the patient's overall condition, and the quality of perioperative care impact the prevalence and incidence rates. Multiple extensive epidemiological studies have examined the frequency of postoperative problems in various surgical fields. These studies frequently employ databases, registries, and electronic health records (EHRs) to encompass a wide range of surgical patients [14]. Analyses of national databases in several regions, including the United States and Europe, have revealed prevalence rates between 10% and 25%. These rates vary depending on the specific surgical operation and the characteristics of the patients. Incidence rates, typically measured as the number of occurrences per 100 or 1,000 surgical procedures, offer a dynamic viewpoint on the likelihood of experiencing complications [15]. Prospective cohort studies and systematic reviews provide valuable data for estimating incidence rates, encompassing immediate postoperative problems and those that arise in the days or weeks after surgery. These rates function as a standard for assessing the efficacy of preventive strategies and interventions designed to decrease complications [15].

Challenges in the Field of Epidemiology

Although epidemiological studies provide crucial data, they also encounter obstacles affecting the precision and applicability of their conclusions. Differences in how postoperative complications are defined and categorized across various studies can result in inconsistencies in the reported rates of occurrence and frequency. Furthermore, there is a possibility of underreporting and poor recording in healthcare records, which can compromise the credibility of epidemiological data [16]. The intricacy of patient populations and the variety of surgical procedures also confound the interpretation of epidemiological findings. Some surgical methods have an inherent increased likelihood of problems, and patient characteristics such as age, comorbidities, and socioeconomic position contribute to differences in results. Acknowledging these difficulties is crucial to correctly evaluate epidemiological data and effectively apply the effects to clinical practice [17]. Identifying risk factors for postoperative problems is essential to categorize risk, provide preoperative guidance, and implement specific therapies. Multiple factors contribute to the heightened susceptibility to problems, including variables relating to the patient, the surgical procedure, and the postoperative period. Considerations associated with the patient include age, prior medical issues, dietary status, and lifestyle considerations [18]. Advancing age is continuously linked to an increased likelihood of difficulties since the body's physiological reserves tend to diminish with the aging process. Coexisting medical illnesses, such as diabetes, cardiovascular diseases, and respiratory conditions, enhance the likelihood of experiencing difficulties [19].

Surgical considerations include the nature and intricacy of the operation, the length of time it takes to perform, and the level of expertise the surgeon possesses. Surgeries that are considered high risk, such as major abdominal or cardiac procedures, naturally have a higher probability of problems [20]. Extended duration of surgery is frequently associated with a heightened risk, underscoring the significance of proficient and accurate surgical techniques. Postoperative variables, such as the level of postoperative care, adherence to guidelines, and early complications, also impact overall results. Thorough monitoring after surgery, prompt identification of difficulties, and suitable therapies help reduce the harmful effects of adverse occurrences [21]. A comprehensive and complex strategy is necessary to effectively deal with the high occurrence of postoperative complications and reduce the impact of related risk factors. Comprehensive preoperative assessment, which includes a detailed examination of the patient and the categorization of risks, allows for personalized care plans and well-informed decision-making [22]. Reducing the overall risk of complications can be achieved by optimizing modifiable risk factors, such as quitting smoking, maintaining glycemic control, and providing nutritional support.

Enhancing the standard of surgical care is crucial in reducing postoperative complications. This entails following evidence-based methods, implementing standardized surgical protocols, and providing continuous education and training for healthcare workers [22]. Improved communication and coordination among diverse teams of surgeons, anesthesiologists, nurses, and other allied health professionals offer a complete approach to patient care. Implementing perioperative care pathways and enhanced recovery after surgery procedures have demonstrated the potential to decrease the occurrence of problems [23]. These paths consist of evidence-based therapies to optimize care before, during, and after surgery. Enhanced recovery principles prioritize the reduction of postoperative stress, the encouragement of early mobilization, and the decrease in opioid usage, which ultimately leads to improved patient outcomes [23]. Gaining knowledge on the frequency and occurrence rates of postoperative complications and identifying the variables that contribute to them is crucial for improving patient care and guiding healthcare practices. Epidemiological studies offer valuable insights into the distribution and causes of problems, informing attempts to improve perioperative care and decrease adverse occurrences [22]. Tackling the difficulties associated with epidemiological research and adopting thorough measures to reduce risk factors are critical variables in the continuous progress of surgical treatment. Continual research and collaboration are essential for developing preventative measures and enhancing the safety of surgical treatments in response to the changing healthcare landscape [24].

Identification and diagnosis of postoperative complications

Identifying and diagnosing postoperative problems are crucial to patient care, enabling prompt management and enhancing outcomes [24]. This discussion will explore the various methods used to identify and diagnose postoperative issues. It will cover clinical assessment, imaging techniques, diagnostic tools, and the significance of biomarkers.

Clinical Assessment

Clinical assessment is a crucial step in detecting postoperative problems. It comprehensively reviews the patient's physical state, vital signs, and clinical symptoms. Postoperative complications may present in diverse manners, and a thorough clinical evaluation helps identify minor alterations that suggest underlying problems [24]. Vital signs, such as heart rate, blood pressure, respiration rate, and temperature, offer crucial data regarding the patient's hemodynamic stability. Indications of problems, such as infection or bleeding, may be observed through persistent tachycardia, hypotension, or fever. Moreover, evaluating the intensity of pain, the degree of respiratory exertion, and the cognitive condition enhances a comprehensive comprehension of the patient's condition after surgery [25]. Physical examination entails carefully observing surgical incisions to detect any indications of infection, hematoma, or dehiscence. Examining the belly by touch might detect swelling, sensitivity, or unusual growths, which can give information about issues within the abdomen. Respiratory auscultation assists in recognizing matters connected to the lungs [26]. In contrast, neurologic tests help detect alterations in consciousness or focused impairments. Clinical assessment encompasses the entire healing trajectory beyond the immediate postoperative phase. Regular surveillance of fluid equilibrium, electrolyte concentrations, and urine excretion assists in detecting issues such as renal impairment or fluid imbalances [27]. Consecutive evaluations enable the timely identification of difficulties, enabling quick intervention and reducing negative consequences.

Imaging and Diagnostic Tools

Imaging and diagnostic tools are essential for identifying and diagnosing postoperative difficulties. They provide precise anatomical information and help visualize potential issues. Various imaging techniques depend on the probable problem and the specific anatomical area being examined. Plain radiographs are frequently employed to evaluate alterations in the thoracic or abdominal regions [28]. Chest X-rays can identify atelectasis, pneumothorax, or infiltrates. In contrast, abdominal X-rays can detect intestinal obstruction or the presence of free air, indicating perforation. Ultrasonography is a powerful method for assessing abdominal and vascular problems. Abdominal ultrasound can detect the presence of fluid collections, abscesses, or vascular anomalies [29]. Doppler ultrasound facilitates the evaluation of blood circulation and the detection of thromboembolic occurrences. Computed tomography (CT) scans offer detailed cross-sectional images and are especially valuable for detecting problems in the abdomen, chest, and head [29]. CT angiography is utilized to evaluate vascular issues. At the same time, contrast-enhanced CT scans aid in detecting abscesses or collections [29].

Magnetic resonance imaging (MRI) is utilized when a more comprehensive soft tissue assessment is necessary. It helps evaluate neurological disorders, musculoskeletal problems, and anomalies in soft tissues. Endoscopy is a medical procedure that observes the gastrointestinal tract, respiratory system, or other interior tissues [30]. They are crucial in identifying problems such as anastomotic leakage, gastrointestinal bleeding, or airway anomalies. These imaging techniques provide a thorough diagnostic approach, allowing healthcare professionals to visualize and define postoperative problems correctly [31].

Role of Biomarkers

Biomarkers have a growing importance in promptly detecting and diagnosing postoperative problems. These molecular or biochemical markers offer unbiased data on physiological processes, enabling the timely identification of the issues. Multiple biomarkers are pertinent in the setting of postoperative problems [32].

C-reactive protein (CRP): Increased levels of CRP are a reliable indication of inflammation and are frequently employed as an indicator for infection problems. Regularly monitoring CRP levels can help track the effectiveness of treatment and the reduction of inflammation.

Procalcitonin (PCT): PCT is a highly responsive indicator of bacterial infections [33]. Monitoring PCT levels aids in distinguishing infectious problems from other sources of inflammation, hence informing decisions regarding the commencement or continuation of antimicrobial therapy.

Troponin: Elevated levels of troponin indicate damage to the heart muscle. They can be a sign of cardiac issues such as a heart attack or stress on the heart during the period around a surgical procedure.

D-dimer: Elevated D-dimer levels indicate the existence of thromboembolic events [34]. Assessing the risk of venous thromboembolism, a common complication after surgery, is beneficial through monitoring D-dimer levels.

Lactate: Increased lactate levels may suggest reduced blood flow to tissues. They can serve as an indicator of systemic consequences such as sepsis or insufficient oxygen supply [35].

Incorporating biomarkers into clinical practice improves the accuracy of diagnosis and enables a more detailed comprehension of postoperative problems. Continual assessment and analysis of biomarker levels over time aid in the continuous surveillance and direct the treatment of the issues [36].

Challenges and Solutions in Identification and Diagnosis

Despite the progress in clinical assessment, imaging, and biomarker application, difficulties continue to exist in identifying and diagnosing postoperative problems. The diagnosis process is complicated by the variability in how individual patients respond, the intricate nature of surgical procedures, and the possibility of symptoms overlapping between distinct issues [37]. Understanding diagnostic findings necessitates a multidisciplinary methodology, which entails cooperation among surgeons, anesthesiologists, radiologists, and laboratory specialists. Proficient clinical assessment, extensive expertise, and proficient intercommunication among healthcare professionals are imperative in successfully managing the intricacies of postoperative care [38]. Integrating EHRs with decision support systems can enable the smooth integration of diagnostic information into the patient care continuum. Uniform standards for postoperative monitoring and diagnostic algorithms enhance uniformity in medical practice, assisting healthcare providers in promptly identifying and addressing issues [39].

Recognizing and determining postoperative complications requires a thorough and multifaceted approach. The combination of clinical assessment, imaging modalities, and biomarkers provides a comprehensive insight into a patient's condition, allowing for the early detection of problems and prompt action [40]. Continuous research, technological progress, and collaboration among healthcare experts are crucial for enhancing diagnostic methods and achieving better patient outcomes in the ever-changing field of postoperative care.

Contributing factors to postoperative complications

The prevalence of postoperative complications is impacted by many factors, including patient-related, surgical, anesthetic, and systemic aspects. Identifying and dealing with these factors is crucial for successfully preventing and managing complications [41]. This debate offers a comprehensive analysis of the variables contributing to postoperative complications, the difficulties in managing them, and the existing practices and guidelines in the Challenges and Solutions in Postoperative Complications. Patient-related factors influence the probability of postoperative complications [42]. Age, preexisting medical issues, nutritional status, and lifestyle choices affect an individual's general health and resilience. Advanced age is frequently linked to reduced physiological reserves, rendering senior patients more vulnerable to problems [22]. Chronic ailments such as diabetes, cardiovascular diseases, and respiratory disorders can heighten the likelihood of unfavorable incidents occurring during the perioperative period. The complexities of the surgical procedure directly influence the results after the operation [24]. The risk of complications is influenced by factors such as the surgeon's expertise and experience, adherence to evidence-based techniques, and careful attention to hemostasis and tissue management. Insufficient surgical procedures can result in problems such as tissue damage, impaired wound healing, and heightened vulnerability to infections [25].

Anesthetic treatment plays a crucial role in determining the outcomes of a surgery. Elements such as the selection of anesthesia, monitoring during the surgery, and care provided before and after the procedure all play a role in the overall health and comfort of the patient. Complications can arise because of insufficient pain management, adverse reactions to anesthetics, and problems associated with airway control [26]. System factors refer to elements associated with the healthcare system and the larger organizational setting. The quality of care offered can be influenced by factors such as the availability of resources, staffing numbers, and workflow efficiency [28]. Insufficient evaluation before surgery, ineffective communication between healthcare teams, and the absence of defined protocols contribute to systemic difficulties that make patients more likely to experience complications [29].

Challenges in managing postoperative complications

The delayed identification of surgical complications presents considerable difficulty in effectively managing them. Subtle clinical indications may be disregarded or incorrectly attributed to the expected surgical recovery process, resulting in delays in identifying and addressing the issue [30]. Delays in recognizing complications are caused by insufficient monitoring, inconsistencies in healthcare professional alertness, and communication obstacles specific to the patient. The lack of protocols for postoperative care impedes uniformity in clinical practice [31]. Differences in care methods among healthcare practitioners and institutions can result in discrepancies in preventing and managing complications. Standardized guidelines enhance a systematic approach, enabling prompt detection of issues and guaranteeing consistency in patient care. Communication barriers hinder the smooth care coordination across healthcare teams, making effective communication crucial [32]. Obstacles to touch, such as insufficient handovers, limited interprofessional collaboration, and ineffective information transfer, hinder the prompt recognition and treatment of difficulties. Practical and concise communication is essential for transmitting vital information and guaranteeing seamless care provision. Resource limits, such as a shortage of people, insufficient equipment, and financial limitations, provide difficulties in effectively addressing postoperative problems [34]. These limitations can influence the accessibility of essential medical resources, cause delays in diagnostic examinations, and impact the overall standard of healthcare. Efficient utilization of resources and strategic allocation are crucial for minimizing the effects of limitations on managing complications [35].

Current practices and guidelines

Contemporary approaches to preventing and managing postoperative complications are based on well-established principles and optimal practices. The World Health Organization (WHO), the American College of Surgeons (ACS), and specialty-specific associations have established guidelines to standardize perioperative treatment and improve patient safety [34]. The guidelines encompass several areas, such as preoperative evaluation, surgical methods, anesthesia administration, and postoperative surveillance. Best practices prioritize a multidisciplinary approach, highlighting the importance of teamwork among surgeons, anesthesiologists, nurses, and other healthcare providers [35]. Preoperative optimization, risk assessment, and adherence to evidence-based methods are essential to optimal procedures. Enhanced recovery after surgery guidelines and integrating therapies based on evidence across the entire perioperative process have demonstrated the potential to decrease complications and accelerate healing [41]. Quality improvement programs aim to evaluate and develop the care processes to improve patient outcomes. These activities encompass continuous monitoring, data analysis, and feedback methods to pinpoint areas that require enhancement. Implementing surgical quality improvement initiatives and engaging in national and international quality registries help create standards and continuously improve treatment procedures [42].

Technological advancements such as EHRs, telemedicine, and health information exchange facilitate efficient communication and information sharing among healthcare teams [33]. Access to data in real-time and technologies that assist decision-making contribute to the early detection of difficulties and promote evidence-based decision-making [34]. To effectively address postoperative complications, it is crucial to thoroughly grasp the variables that contribute to them, recognize the problems they present, and follow the current practices and standards for their management [34]. Implementing a patient-centered, multidisciplinary approach incorporating the best methods, standardized protocols, and quality improvement programs is essential in optimizing postoperative treatment and increasing outcomes. Continual research, technological advancements, and cooperation among healthcare professionals contribute to developing solutions to tackle the ever-changing difficulties related to postoperative complications [35].

Innovative approaches and technologies

Over the past few years, new and creative methods and technology have been crucial in changing how surgical care is provided. The goal is to tackle difficulties and improve patient results regarding postoperative problems [36]. This discussion examines different aspects of innovation, including progress in surgical methods, the influence of robotics and minimally invasive surgery, digital health solutions, interdisciplinary collaboration, team-based approaches, communication among healthcare professionals, patient education and engagement, and strategies for patient empowerment and compliance with postoperative instructions.

Progress in Surgical Techniques

The area of general surgery has been dramatically impacted by advancements in surgical techniques, which have provided novel answers to long-standing issues. Contemporary surgical methods promote accuracy, effectiveness, and minimized invasiveness to improve patient recovery [35]. Methods like laparoscopy and robotic-assisted surgery are prime examples of this fundamental change in approach. Laparoscopic techniques entail the utilization of specialized equipment and tiny incisions, resulting in reduced tissue stress and expedited postoperative recovery. Robotic-assisted surgery merges the accuracy of robots with the surgeon's expertise, allowing for enhanced agility in intricate procedures [35]. These technological improvements play a role in diminishing postoperative discomfort, shortening hospital stays, and accelerating the resumption of regular activities, thereby reducing the likelihood of problems.

Role of Robotics in Minimally Invasive Surgery

Robotic-assisted surgery and minimally invasive procedures are revolutionary advancements in the field of surgery. Robotics, specifically, have advanced to become essential in multiple surgical disciplines [35]. The da Vinci surgical system (Intuitive Surgical, Sunnyvale, California, United States) allows surgeons to do intricate procedures with heightened accuracy and command using robotic arms manipulated by a console. Minimally invasive surgery, such as laparoscopy, endoscopy, and arthroscopy, minimizes surgical damage and expedites patient recuperation [37]. These methods lead to more minor cuts, reduced bleeding, and less pain after surgery, all of which contribute to a lower chance of complications than traditional open surgery.

Technological Healthcare Solutions

Incorporating digital health solutions into postoperative care has provided innovative methods to monitor and manage patients remotely. Telehealth systems, wearable gadgets, and mobile applications enable instantaneous connections between patients and healthcare providers [11]. Remote patient monitoring enables the continuous monitoring of vital signs, activity levels, and other pertinent parameters. Digital health technologies improve postoperative monitoring and empower patients with self-management tools and educational resources [14]. This technology-driven strategy enhances the availability of healthcare services, facilitates early identification of issues, and cultivates a cooperative alliance between patients and their healthcare providers.

Multidisciplinary collaboration

Postoperative treatment is intricate and requires a multidisciplinary approach involving cooperation among different healthcare specialists. Surgeons, anesthesiologists, nurses, physical therapists, dietitians, and other professionals collaborate to enhance patient outcomes through their expertise [15]. Collaboration across multiple disciplines guarantees a thorough assessment of patient requirements, enables the prompt detection of possible difficulties, and promotes synchronized interventions [17]. Team-based care models facilitate efficient communication, collaborative decision-making, and smooth patient transitions throughout their surgical experience.

Significance of Collaborative Approaches

Team-based approaches to postoperative care recognize that the success of surgical interventions extends beyond the operating room. The cooperation of healthcare professionals, encompassing surgeons, anesthesiologists, nurses, and rehabilitation specialists, is crucial in delivering comprehensive care [18]. Collaborative decision-making, frequent interdisciplinary meetings, and transparent communication channels enhance a patient-centered approach. This collaborative paradigm improves the standard of treatment, encourages consistency, and caters to the varied requirements of patients, thus decreasing the likelihood of complications and enhancing overall results [19].

Interprofessional Communication in Healthcare

Efficient communication among healthcare workers is crucial for achieving successful postoperative care. Practical and concise communication guarantees the accurate transmission of vital information, minimizing the probability of mistakes and omissions [20]. Structured handovers, frequent team meetings, and defined communication procedures all contribute to forming a unified healthcare team. Interprofessional education and training programs bolster communication skills, cultivating a culture of collaboration and mutual esteem [22]. Efficient and precise communication between healthcare personnel allows for quick identification of issues. It permits speedy intervention, reducing the likelihood of negative consequences.

Education and engagement of patients

Enabling patients via education and active participation in their healthcare is a fundamental principle of contemporary healthcare. Regarding postoperative problems, patient education is crucial in preparing individuals for surgery, establishing realistic expectations, and encouraging compliance with postoperative recommendations [24]. By offering lucid and easily understandable details regarding the surgical operation, potential problems, and the significance of postoperative care, patients are empowered to engage in their recuperation process actively [25].

Approaches to Enhance Patient Empowerment

Educating patients about the surgical process, expected outcomes, and potential issues before the procedure helps their understanding and minimizes anxiety. Preoperative educational workshops, informative brochures, and multimedia materials facilitate the establishment of realistic expectations and encourage well-informed decision-making [26]. Adapting care plans to every patient's distinct requirements and desires promotes empowerment. Patient participation and adherence to the suggested postoperative regimen can be enhanced by providing tailored instructions, rehabilitation plans, and nutritional advice [28]. Health literacy initiatives are crucial for effectively empowering patients by addressing problems related to health literacy. Utilizing precise and concise language, visual aids, and interactive tools enhances patients' understanding of medical information, empowering them to make well-informed decisions and actively engage in their healthcare [29]. Engaging patients in shared decision-making processes enables them to participate actively in decisions regarding their care. Engaging in a conversation about treatment alternatives, possible hazards, and anticipated results empowers patients to make decisions that match their personal beliefs and preferences [30].

Improving Patient Compliance with Postoperative Instructions

Strict compliance with postoperative instructions is essential for successful recovery and minimizing the risk of complications. Multiple ways contribute to improving patient adherence [12]. Offering unambiguous, concise, and readily understandable postoperative instructions aid patients in understanding their obligations throughout the recuperation phase. Utilizing digital platforms and mobile applications for postoperative education and communication promotes continuous involvement [9]. Patients' dedication to their treatment plans is strengthened by including interactive elements, reminders, and the ability to track their progress. Multimodal communication involves using different communication channels, such as textual materials, vocal instructions, and visual aids, to accommodate different learning styles and ensure that patients receive information in a way that connects them [8]. By implementing comprehensive follow-up mechanisms such as scheduled postoperative checkups, remote monitoring, and telehealth consultations, healthcare providers can effectively evaluate patient progress, address concerns, and encourage adherence to instructions [10].

Novel methodologies and advanced technologies are transforming the postoperative care domain, providing remedies to obstacles and enhancing patient results [3]. Various innovations, including advancements in surgical techniques, robotics, digital health solutions, multidisciplinary collaboration, team-based approaches, effective communication among healthcare professionals, patient education, and strategies for empowerment and adherence, enhance the comprehensive and patient-centered model of postoperative care [4]. Incorporating these novel methods into regular clinical processes shows excellent potential for tackling the difficulties of preventing and managing postoperative complications.

Quality improvement initiatives

Postoperative care quality improvement programs are essential for improving patient outcomes, reducing complications, and optimizing healthcare delivery. This conversation explores many aspects of quality improvement projects, such as continuous quality monitoring, benchmarking, and auditing [13]. Furthermore, it delves into prospective avenues and topics of study, embracing subjects that require additional examination and developing technologies and therapies within the framework of Challenges and Solutions in Postoperative Complications. Continuous quality monitoring refers to the ongoing process of systematically assessing and evaluating the quality of a product or service [16] and entails the systematic and continual assessment of healthcare procedures. It results in pinpointing areas that can be enhanced. Continuous monitoring is crucial in postoperative care to identify deviations in practice, evaluate the effectiveness of interventions, and guarantee the provision of high-quality, evidence-based care [23]. Comprehensive monitoring systems incorporate diverse variables such as complication rates, adherence to clinical guidelines, patient satisfaction, and resource use. The progress in EHRs has enabled real-time monitoring systems. Embedded inside EHRs, automated triggers and alerts can promptly warn healthcare practitioners of deviations from established norms [29]. This allows for early interventions and corrective actions to be taken. Continuous quality monitoring offers valuable insights into the efficacy of existing procedures. It serves as a basis for evidence-based enhancements in postoperative care [30].

Comparative Analysis

Benchmarking entails evaluating an organization's performance indicators about those of its peers or established benchmarks. Within the realm of postoperative care, benchmarking assists healthcare organizations in identifying their areas of excellence and regions needing improvement [31]. Comparisons can be made regarding complication rates, patient satisfaction scores, length of hospital stays, and other pertinent outcome measures. Healthcare quality organizations and professional associations administer national and worldwide databases that provide essential benchmarking data [32]. Institutions can employ benchmarking to establish attainable performance objectives, monitor advancements over time, and incorporate superior methodologies from top-performing counterparts. Healthcare practitioners can utilize benchmarking data to strategically deploy treatments that effectively minimize disparities in care and improve overall quality [35]. Auditing refers to the systematic examination and evaluation of financial records, statements, and transactions to ensure accuracy, compliance with regulations, and fraud detection. Auditing entails examining healthcare systems and activities to verify adherence to standards and norms. Postoperative care auditing evaluates compliance with clinical protocols, surgical safety checklists, and quality indicators [36]. Regular audits facilitate the detection of areas that need improvement, the assessment of resource allocation, and the strengthening of a safety-oriented culture [40].

Internal and external audits have interdependent functions in the auditing process. Healthcare organizations undertake internal audits to assess their internal processes, protocols, and compliance with specified standards [41]. External audits, typically conducted by regulatory organizations or certifying agencies, offer an extra level of examination and guarantee compliance with broader industry norms [42]. Audits provide valuable information that informs attempts to improve quality and contribute to the continuous improvement of postoperative care procedures.

Future directions and research needs

Subsequent studies on postoperative care should delve into intricate facets of complications, patient outcomes, and healthcare processes. Potential areas for additional research are the correlation between socioeconomic characteristics and rates of complications, the efficacy of initiatives aimed at mitigating health inequities, and the impact of patient engagement techniques on outcomes following surgery [12]. Other areas to consider are exploring the long-term repercussions of surgical problems and the economic ramifications of preventive interventions. Enhancing the models used to stratify risk is crucial for identifying individuals at a greater risk of experiencing problems [14]. The research should prioritize enhancing predictive models by integrating innovative biomarkers, genetic factors, and machine learning algorithms. Improved risk assessment allows for focused interventions, customized care plans, and efficient allocation of resources [16]. Ongoing research should focus on investigating the most effective protocols for perioperative care, encompassing preoperative optimization, intraoperative treatments, and postoperative management methods. Examining the effects of enhanced recovery after surgery protocols and multidisciplinary care models on complication rates and recovery trajectories would help improve perioperative care standards [17].

Incorporating patient-reported outcomes (PROs) into studies on postoperative treatment is becoming increasingly important. Subsequent research should investigate the association between PROs and conventional clinical measures to better comprehend patient experiences and quality of life after surgery [20]. This strategy improves the emphasis on patient-centeredness in postoperative care research.

Advancements in Technologies and Therapies

The incorporation of telehealth and remote monitoring technology can transform postoperative treatment. Research should investigate the effectiveness of virtual follow-up visits, wearable devices, and mobile applications in promoting early identification of problems, strengthening patient compliance with postoperative instructions, and improving overall recovery [22]. The progress made in precision medicine, which involves analyzing genetic characteristics and identifying biomarkers, presents possibilities for tailored interventions [24]. Research should explore the impact of precision medicine on customizing treatment plans, forecasting individual reactions to drugs, and reducing the likelihood of problems by considering genetic and molecular characteristics. Further investigation into robotic-assisted surgery and augmented reality technology use is crucial [30]. An examination of the effects of these technologies on surgical accuracy, results, and rates of complications will aid in their incorporation into regular clinical procedures. Pharmacogenomic research can provide insights into individual differences in drug metabolism and response, which can impact the choice and dosage of medications [36]. Furthermore, investigating innovative therapeutic techniques, such as precise administration of drugs and regenerative medicine methods, shows potential for reducing problems and enhancing postoperative recovery.

Continuous quality monitoring, benchmarking, and auditing are essential elements of quality improvement efforts in postoperative care, serving as a basis for evidence-based practice and systematic enhancement of healthcare delivery. As we go forward in postoperative care, the need for research and the emergence of new technology offer chances to improve patient outcomes, optimize perioperative care, and investigate new therapeutic methods. To tackle the issues related to postoperative complications and advance the field of surgical care, the healthcare community can promote innovation, collaboration, and continuous research.

## Conclusions

Effectively dealing with the difficulties presented by postoperative complications requires a thorough and proactive approach based on ongoing efforts to enhance quality. Incorporating technology such as telehealth, precision medicine, and robotic-assisted surgery has significant potential for improving patient outcomes. The focus on patient-centered treatment, supported by teamwork among multiple disciplines and efficient communication, highlights the dedication to overall well-being. Continued research and exploration of new technologies will shape the future of postoperative care. This will create a healthcare system where complications are reduced, patients are empowered, and surgical practice focuses on achieving excellence.
